# Pragmatism as Idealism? The Case of Mary Whiton Calkins

**DOI:** 10.1002/jhbs.70028

**Published:** 2025-06-01

**Authors:** Lisa Kampen

**Affiliations:** ^1^ Center for the History of Philosophy and Science, Faculty of Philosophy, Theology and Religious Studies Radboud University Nijmegen the Netherlands

**Keywords:** American pragmatism, idealism, Mary Whiton Calkins, self‐psychology, William James

## Abstract

American pragmatism is traditionally described as a logico‐philosophical movement that arose in opposition to the theological and metaphysical assumptions of the early American idealists. Mary Whiton Calkins (1863–1930) challenges this narrative in two central ways: she presents pragmatism as encompassing psychological, logical, and metaphysical doctrines, and she characterizes “metaphysical pragmatism” as a form of idealism. This paper offers a systematic reconstruction of Calkins's philosophical and psychological commitments to explain these challenges to the traditional narrative. The first part proposes that Calkins's taxonomy of “pragmatisms” reflects her views on the proper roles of philosophy and psychology. During a time when psychology was separating from philosophy, Calkins argued that psychology should study reality as experienced through introspection, while philosophy should address the metaphysical nature of the realities experienced. The second part of this article explores Calkins's rationale for characterizing metaphysical pragmatism as idealism. She understood the pluralism characteristic of pragmatism as a “pluralistic personalism”, where reality is the nature of consciousness, and consciousness is viewed as a plurality of conscious selves. Calkins rejected metaphysical pragmatism because she believed that pluralism could not account for absolute truth. However, she maintained support for psychological pragmatism, believing it to be metaphysically neutral. The article concludes with a brief critical analysis of Calkins's position on pragmatism as regards her philosophy‐psychology distinction and the supposed opposition between pragmatism and metaphysics more broadly.

## 1 Introduction

In 1908, 10 years after William James (1842–1910) had introduced the term “pragmatism” to the Philosophical Union of the University of California in Berkeley, his once student Arthur Lovejoy (1873–1962) wrote an article in which he argued that “it is perhaps not too much to ask that contemporary philosophers should agree to attach some single and stable meaning to the term” (Lovejoy 1908, 5). Lovejoy warned that the ambivalence surrounding the term could serve as a pretext to dismiss pragmatism altogether. At that moment, he believed to be able to identify “thirteen pragmatisms”, all “logically independent, so that you may without inconsistency accept any one and reject all the others” (Lovejoy 1908, 5).

In 1907, a year before Lovejoy's article, another of James's alumni presented a taxonomy of “pragmatisms”, identifying three separate understandings. Her name was Mary Whiton Calkins (1863–1930), and she had just finished her great philosophical treatise, *The Persistent Problems of Philosophy* (1907). In that treatise, Calkins did not argue for a single understanding of pragmatism, but noted that the confusion of “one pragmatism” with others leads pragmatists to reiterate statements that are already accepted by their critics, while failing to effectively defend the maxim that prompted James's lecture in the first place, namely Charles Sanders Peirce's (1839–1914) foundational statement:

Consider what effects, which might conceivably have practical bearings, we conceive the object of our conception to have. Then, our conception of these effects is the whole of our conception of the object.

(Peirce 1878/2011, 46)

At the time Calkins presented her “three pragmatisms,” she was revered mainly as a psychologist, having in 1905 served as the first female president of the American Psychological Association. Calkins had begun her career as an instructor in Greek at Wellesley College, following her master's degree in philosophy and classics from Smith and a period of study in Greece (Furumoto 1979; Dykeman 1993). In the early 20th century, psychology was in the initial stages of becoming an independent scientific discipline, which meant that in most academic institutions, psychology courses were still taught at philosophy departments (Campbell 2006; Pickren and Rutherford 2010; Benjafield 2015). Accordingly, it was the then Professor of Philosophy at Wellesley who proposed that Calkins be offered a position in the department of “Mental Philosophy”, which included both psychology and metaphysics (Calkins 1931, 8–9). Trained a classicist, Calkins initially thought the idea was “preposterous”, but accepted it a few days later on the condition that she be allowed a year off to study psychology in depth (Calkins 1931, 8–9). This resulted in a 5‐year study at Harvard with George Palmer (1842–1933), Josiah Royce (1855–1916), Hugo Münsterberg (1863–1916), and William James. Despite the fact that in these 5 years, Calkins had done all the work required for the Harvard PhD degree, and James later described Calkins's defense as “the most brilliant examination for a PhD that we have had at Harvard”, she was denied the doctorate on the basis of her gender (James 1895; Furumoto 1979; Zedler 1995; McDonald 2006).

The absence of a doctoral degree did not stop Calkins from establishing herself as one of the prominent American philosopher–psychologists at the time, alongside contemporaries such as John Dewey (1859–1952) and George Herbert Mead (1863–1931). After establishing a psychological laboratory at Wellesley, publishing her first psychological handbook, and her election as the first female president of the American Psychological Association, Calkins started to publish more and more in the domain of philosophy, ultimately serving as the first female president of the American Philosophical Association in 1918. She ended her career as a Research Professor of Philosophy and Psychology at Wellesley, with four authored books, four edited volumes, and over a 100 articles published (Service in Memory of Mary Whiton Calkins 1930).

Much like her fellow philosopher–psychologists, Calkins sought to establish psychology as a legitimate science while also defending philosophy as an independent discipline. What makes Calkins's writings on pragmatism unique is that they reflect this effort, as her taxonomy of pragmatisms is based on a disciplinary distinction between psychology, logic, and metaphysics. She believed that “psychological” and “logical pragmatism” were compatible with different metaphysical conceptions of reality, while “philosophical” or “metaphysical pragmatism” necessitated a plural conception of reality (Calkins 1907, 560). To Calkins, the confusion of psychological and logical pragmatism with metaphysical pragmatism had led pragmatists to unnecessarily reiterate statements of the psychological and logical doctrines, while failing to effectively defend the pluralism that was underlying the metaphysical doctrine of pragmatism (Calkins 1907, 559−560).

Another remarkable feature of Calkins's interpretation of pragmatism is that she considered the pluralism of metaphysical pragmatism a type of idealism called “pluralistic personalism”. According to her, pluralistic personalism described reality as “of the nature of consciousness” (Calkins 1907, 406). More specifically, the pluralistic personalists depicted reality as a collection of selves, stating that consciousness was ultimately “a unique ‘real’ which is conscious and […] more permanent than ideas are” (Calkins 1907, 406–407).

These views by Calkins challenge the traditional historical narrative on pragmatism in two critical ways. The first is that pragmatism is usually considered a logico‐philosophical movement, in which Peirce focused more narrowly on logic, while James interpreted pragmatism more broadly as “a new name for some old ways of thinking” (Peirce 1878/2011; James 1907). Calkins challenged this logico‐philosophical understanding with the argument that pragmatism involved three distinct doctrines: psychological, logical, and metaphysical, in which the emphasis lay on the psychology–metaphysics distinction. My explanation will focus on this distinction and propose that it reflects Calkins's views on the appropriate subject matters and methodologies of psychology and philosophy, respectively.

The second way in which Calkins challenges the historical narrative is by characterizing metaphysical pragmatism as a form of idealism. Let us recall that pragmatism may be retraced to the members of the “Metaphysical Club”, among them Peirce and James. The Metaphysical Club was an ironically misnamed reading group founded at Harvard in 1871, which had the aim to break away from the metaphysical import of the American idealists (Menand 2001; Misak 2013; Shook 2019). Idealism had become dominant at American universities during the 19th century, following the influence of Berkeley and the German Idealists in Europe (Shook 2019; Verhaegh In press). While scholars such as Cheryl Misak are careful to depict the thought of the members of the “Metaphysical Club” as a navigation between the empiricism of Locke and Hume and “the strong theological or (in the parlance of the time) ‘metaphysical’ assumptions of the early American idealists”, others, such as Charlene Haddock Seigfried, insist that it is a “longstanding misconception” to understand pragmatism as the continuation of the American idealist tradition (Seigfried 2001, 13; Misak 2013, 8).

My analysis of the two ways in which Calkins challenges the traditional pragmatism narrative will be structured into four sections. Given the fact that Calkins is still under‐researched, Section 2 provides a contextualization of her ideas during the period in which psychology and philosophy were establishing themselves as separate academic disciplines. Section 3 addresses the first way in which Calkins challenges the pragmatism narrative by examining how her ideas on the distinction between psychology and philosophy underpin her taxonomy of the different pragmatisms. Section 4 focuses on the second way in which Calkins challenges the pragmatism narrative, by analyzing her characterization of metaphysical pragmatism as a type of idealism and explaining how Calkins considered some of the pragmatists to have acknowledged free willing selves as the most basic fact of reality. Finally, Section 5 reflects on Calkins's partial endorsement of pragmatism in her later writings, and how her understanding of reality as thoroughly personal and mental in nature influenced this perspective. I conclude by stating that Calkins's writings on pragmatism reveal psychological and philosophical commitments that raise interesting questions for her separation between psychology and philosophy, as well as the relation between pragmatism and metaphysics more broadly.

## 2 Mary Whiton Calkins, the Philosopher–Psychologist

Calkins proposed her disciplinary distinction between psychological, logical, and metaphysical pragmatism within the context of important institutional developments in American academia. During the late 19th century, the new science of psychology had established itself by separating from philosophy. At the same time, philosophers were urged to reidentify their discipline, following the transition of American universities from theological institutions into professional centers of research (Campbell 2006; Verhaegh In press). Central to both developments was Calkins's Harvard mentor James, the same man who also introduced the term “pragmatism” to the Philosophical Union of the University of California in Berkeley (Kuklick 2001; Leary 2009).

James depicted the state of psychology at the beginning of the 20th century as “only the hope of a science”, because according to him, the new science of psychology did not yet “stand on solid ground”, so that its “elementary assumptions and data must be reconsidered” (James 1892b, 335). With James, contemporary philosopher‐psychologists almost unanimously agreed that psychology and philosophy should be separated, but how this was to be done was a topic of heated discussion. In this section, I study this discussion to introduce and contextualize Calkins's ideas on the distinction between psychology and philosophy, so that in the subsequent section, we might see how this disciplinary distinction underlies her taxonomy of pragmatisms.

### 2.1 Structuralism and Functionalism

One of the central considerations in establishing psychology was determining the appropriate methodology. Many argued that for psychology to be recognized as a science, it should adopt the empirical methods of observation and experimentation used in the natural sciences (Schultz and Schultz 2004; Mandler 2007). However, unlike the natural sciences, psychology focused on inner experiences—thoughts and feelings—rather than the physical phenomena studied in fields like physics or chemistry (Campbell 2006; Pickren and Rutherford 2010). The dominant positions with respect to how these psychical phenomena should be studied were offered by two psychological camps, the structuralists and the functionalists.

The structuralists were led by Edward Bradford Titchener (1867–1927), who especially in his early work, argued that the methodology of psychology was the same as that of the other sciences (Green 2010; Evans 2012). His handbook, *A Beginner's Psychology* (1915), states that the method of all sciences “may be in a single word summed up as observation” (Titchener 1915, 19). Although the type of experience that psychology focused on was a “mental experience”, its observation did not for that matter differ from that of physical phenomena, as both concerned “a certain attitude towards phenomena, a vivid experience of the particular phenomenon which is the object of observation, and an adequate report of this experience in words” (Titchener 1915, 19). This meant that psychology had recourse to experiment, which “is simply an observation that may be repeated, that may be isolated, and that may be varied” (Titchener 1915, 23). Through experiment, the structural psychologist is tasked with analyzing complex mental states into simple elements of consciousness. For example, the complex sensation of a needle that pierces a hand can be described by the simple elements “pressure, cold, warmth and pain” (Titchener 1915, 44). In one of his first descriptions of structural psychology, Titchener compared the task of the psychologist to vivisection:

His task is a vivisection, but a vivisection which shall yield structural, not functional results. He tries to discover, first of all, what is there and in what quantity, not what it is there for.

(Titchener 1915, 450)

Opposing this structural approach to psychology, the functionalists argued that the primary focus of psychology should be on the functions of consciousness rather than on “what is there”. Stated in Titchener's terms, their idea was that the description of “what is there” always rests on “what it is there for” (Titchener 1915, 450). So, whereas structuralists wanted to find out what consciousness consisted of to understand how it functioned, functionalists such as Dewey or James Mark Baldwin (1861–1934) pointed out that the functions of consciousness *determine* what consciousness consists of (Dewey 1896; Baldwin 1902). For example, Dewey argued that “sensory stimulus, central connections and motor responses” should not be viewed “as separate and complete entities in themselves, but as divisions of labor, functioning factors, within the single concrete whole” (Dewey 1896, 361). Because the elements of consciousness are functioning factors rather than entities, the functionalists stressed that their isolation in psychological experiments only served as an idealization rather than being an accurate psychological description (Dewey 1896, 1920). Functionalists were often also pragmatists, although functionalism was not exhausted by pragmatism. The “functional pragmatists” claimed that their focus on the functions of consciousness went hand in hand with an open‐ended or “non‐complete” conception of reality (Dewey 1920). As we will see in the next section, Calkins considered this type of reasoning an example of the confusion of one doctrine of pragmatism with another.

It should be noted that in practice, the structuralists and the functionalists differed less from one another than this brief description might suggest. In fact, Calkins's early writings on psychology were inspired by the fact that many psychologists oscillated between the two methods without proper explanation. For example, she pointed out that:

The common procedure, it must be confessed, is the confusion of the two points of view and the oscillation from one method to another. Wilhelm Wundt, G.T. Ladd, Harold Höffding and William James are representatives of this tendency.

(Calkins 1901, 445–446)

To strengthen her point, Calkins emphasized that the German “Father of Psychology”, Wilhelm Wundt (1832–1920), was studying certain mental processes via the structural method, such as percepts and images, while he investigated others, such as the will, as functions (Calkins 1901, 445–446). She stressed that this oscillation between the two psychological methods resulted in a fragmented division of mental processes that is “as unnecessary as it is misleading” (Calkins 1899, 378). Beginning with her APA address, Calkins proposed a conception of psychology that, in her view, could reconcile structural and functional methods, thereby avoiding the unwarranted division. She named this approach “self‐psychology” (Calkins 1906).

### 2.2 Psychology as Science of Selves

Calkins argued that although we may define psychology “provisionally as science of consciousness,” it is “more exactly” defined “by naming it science of the self as conscious” (Calkins 1910, 1). This statement was inspired by James's claim that the first character of thought is that it belongs to a personal consciousness (Calkins 1930a; Bella 2022). James writes in his *Principles* that “whether anywhere in the room there be a mere thought, which is nobody's thought, we have no means of ascertaining, for we have no experience of its like” (James 1890/1950, Ch. 9, p. 226). Accordingly, Calkins states that “there is never perception without a somebody who perceives, and there never is thinking unless someone thinks” (Calkins 1910, 1).

Calkins argued that the opposition between structuralism and functionalism stemmed from a disagreement over the proper subject matter of psychology, rather than its methodology (Calkins 1906, p. 64). She agreed with the functionalists that structuralism neglected the environment of the individual, but instead of criticizing their methodology, she argued that this resulted from the structuralists' understanding of the basic fact of psychology as consciousness composed of isolated elements (Calkins 1906, 63–65). For Calkins, the opposing understanding of the basic fact of psychology proffered by many functionalists was the psychophysical organism, which she also rejected on the account that it lacked introspective accessibility (Calkins 1906, 63–76). She proposed that if both structuralists and functionalists were to adopt the “self as psychic fact” as the most basic constituent of psychology, their methodologies could be reconciled (Calkins 1906, 63).

By “self as psychic fact”, Calkins explained, “I mean what the plain man means by self” (Calkins 1906, 63). Following her argument that perceptions and thoughts never appear without a perceiver or thinker, she stressed that the self is present in every percept, thought, or other mental state (Calkins 1910, 3). Her arguments were based on introspection, which Calkins believed to be the fundamental method of psychology, one that could be supplemented by physiological experimentation and description (Calkins 1901, 1910, 1930b). She wrote in her autobiography that her emphasis on introspection was inspired by James's *Principles of Psychology*, which includes the claim that “introspective observation is what we have to rely on first and foremost and always” (Calkins 1930a, 31; James 1890/1950, Ch. 7, p. 185). In the *Principles*, James had argued that introspection was almost too straightforward to be defined, since “it means, of course, the looking into our own minds and reporting what we there discover” (James 1890/1950, Ch. 7, p. 185). Calkins provided a similar definition in her first psychological work, *An Introduction to Psychology* (1901), adding that “one has merely to ask oneself such questions as: ‘How do I actually feel?’ ‘What do I mean when I say that I perceive, remember, believe?’” (9).

Michela Bella notes how James's influence on Calkins can be thought of as “a methodological imprinting of the primacy of introspection” (Bella 2022, 71). “Imprinting” seems the correct term, as Calkins defended James's statements on introspection more fiercely than even he himself did (James 1890/1950; Calkins 1901, 1907, 1910). To provide just one example, James on more than one occasion admitted his doubt on whether “we have direct introspective acquaintance with our thinking activity as such”, asking in his *Briefer Course* whether the existence of an inner sense as the subject of experience was not rather a postulate, so that “the question of *who the knower really is* [is thrown] wide open again” (James 1892b, 467). In contrast to James, Calkins treated introspective experiences throughout her work as almost infallibly certain, among which the most important is “the self as immediately experienced” (Calkins 1910, 3).

Calkins believed the introspected self to be implied in every structural explanation (Calkins 1906, 70). She noted that “it is past doubt” that the “structural analysis of a psychic state is always possible”, so that, in Titchener's terms, the experience of a needle that pierces a hand could be described by the elements pressure, cold, warmth, and pain (Calkins 1906, 70; Titchener 1915). What this structural description implied, however, is that it is a self that got her hand pierced, so that was her personal experience of pressure, cold, warmth, or pain. Calkins argued that if the structuralists articulated the self in their sensational descriptions, they would see that the sensations belong to a self that is always in relation to external objects and other selves, such as the needle and needle‐piercer (Calkins 1906, 1910). In other words, the articulation of the self revealed a relation of this self to the environment, so that by acknowledging the self as a basic fact, structural psychology becomes compatible with functional psychology (Calkins 1906).

Yet Calkins also noted that some functional psychologists went one step further by explaining the self as psychophysical (Calkins 1906). She rejected this understanding of the self on the basis that she saw no introspective argument for the amalgamation of psychic and physical realities (Calkins 1905, 1908a, 1910). Calkins wrote that “it may be doubted whether such a conglomerate of psychical and physical does not actually affront the everyday experience which it has claimed as its chief warrant” (Calkins 1905, 266). She believed that although functionalists point to specific psychophysical functions, such as “selection, adaptation and variation”, they “never escape the necessity of distinguishing from these the ‘purely psychical’ and the ‘merely physiological’ functions” (Calkins 1908a, 13). In one of her last writings, Calkins expresses this conviction most emphatically as follows:

I know as immediately that the subvocal contraction of throat‐muscles in pronouncing a word, say ‘justice,’ is a phenomenon distinct from that of ‘thinking’ justice, though the two phenomena are closely correlated.

(Calkins 1930b, 201)

Calkins argued that as long as introspection reveals a gap between physical phenomena, such as the subvocal contraction of throat‐muscles in pronouncing the word “justice” and psychic phenomena such as thinking “justice”, we cannot accept the self as psychophysical. To her, the psychologist must accept “the self as object of introspection, raising no questions about its ultimate reality” (Calkins 1908b, 272).

### 2.3 The Distinction Between Psychology and Philosophy

Calkins's statement about the psychologist's acceptance of the self as “object of introspection” is related to what she considered to be the scope of the science of psychology. Calkins defined science as “a systematic study of facts or phenomena; that is, of limited or partial realities” (Calkins 1901, 4). She explained that a fact possesses three characteristics:

(1) it is one‐of‐many; (2) always a dependent bit of reality, never the self‐dependent whole; and (3) it is taken for granted or assumed, without question of the nature of its reality or of its own relation to the whole‐of‐reality.

(Calkins 1900, 491)

In her philosophical treatise, Calkins argued that this conception of fact follows from any practice of the scientist:

The physiologist, for example, does not inquire whether or not the limited object of his study, the living cell, is in its fundamental nature a physical or psychical phenomenon—whether, in other words, protoplasm reduces, on the one hand, to physical energy, or, on the other hand, to consciousness. On the contrary, the physiologist, properly unconcerned about the completeness or about the utter irreducibleness of his object, confines himself to analysis within arbitrary limits of his living cells, leaving to the philosopher the questions: What is the real nature of these psychical and these physical processes? Is reality ultimately split up into psychical and physical? Is the division a final one, or is the psychical reducible to the physical? Is thought a function of brain activity? Or, finally, is the physical itself reducible to the psychical; that is, is matter a manifestation of conscious spirit?

(Calkins 1907, 5)

For Calkins, a given science may be considered more or less fundamental with respect to whether it takes its facts for granted (Calkins 1900). For example, chemistry is considered more fundamental than physiology, as chemistry analyzes its facts in terms of simpler elements, and physiology generally studies them in relation to a larger whole, the living system. However, she made it clear that even when a science is considered to be fundamental, it does not thereby become philosophy, “until it ceases to deal with the related manifold of fact, and attains the conception of a self‐dependent reality” (Calkins 1900, 491).

Calkins's conception of the limited scope of psychology fits in with the efforts of the early psychologists to separate their discipline from philosophy, and the influence of James might again be noted here (James 1890/1950, 1892b). Moreover, her conception of *philosophy* fits the perceived need of philosophers in the United States of the early 20th century to distinguish themselves from the upcoming scientific disciplines, on the one hand, and the old religious curriculum, on the other (Campbell 2006; Verhaegh In press). Sander Verhaegh explains how, as the previously dominant clerical influence in American academia was waning, philosophers needed to persuade university presidents of the fact that philosophy offered more than theology, while also preventing their discipline from “being swallowed by the sciences” (Verhaegh In press, 9). He emphasizes how the initial answer of American philosophers was to present philosophy as a systematic discipline that “grounded and unified scientific, moral and religious truths” (Verhaegh In press, 9). As we have seen, the type of philosophy that was considered as being up to such a task was generally an idealism in the wake of Berkeley or the German Idealists.

In line with Verhaegh's characterization of the American philosophical landscape, Calkins argued that “there is room for a philosophy fundamental to science, and that it need not be a vague or abstract study” (Calkins 1907, 11). She agreed with traditional conceptions of philosophy as concerned with questions on the nature of reality and stated “unambiguously that by ‘philosophy’ I mean ‘metaphysics’” (Calkins 1930b, 199). She also sided with the German idealists in their critical acknowledgement of Kant, who had rejected a “dogmatic” or rationalist understanding of this metaphysics (Calkins, “Philosophy Notes,” n.d.). From this perspective, Calkins stated that:

philosophy *must* take as its object the utterly irreducible nature of some reality; and philosophy *may* take as its object the ultimate nature not only of a single fact or group of facts, but of all‐that‐there‐is.

(Calkins 1907, 4)

Calkins thought it important that philosophy takes as its object of study “the utterly irreducible nature of some reality”, because she understood this to be the only positive criterion that distinguishes philosophy from science (Calkins 1907, 4). The negative criterion to distinguish science and philosophy was that the latter “does not seek and classify facts, but rather takes its materials ready‐made from the sciences, simply reasoning about them and from them” (Calkins 1907, 4). However, Calkins believed that if this were the only difference between philosophy and science, the former would not be a distinct discipline, as philosophy, “if conceived simply as the process of reasoning about scientific phenomena, would be merely the explanatory side of science” (Calkins 1936, p. 4).

Calkins's thought on the subject matter of philosophy vis‐à‐vis the subject matter of psychology also applies to her concept of self, which is not only central to her psychology but also to her philosophy. She passionately argued that self‐psychology is a legitimate science, as the self of the psychologist is experienced in the same sense as other scientific facts are experienced, such as qualities, events, and things (Calkins 1900, 1901, 1907). The psychologists's self is characterized as one‐of‐many, a bit of reality instead of the self‐dependent whole and taken for granted in every‐day observation (Calkins 1900). The scientific study of the self should also be understood as entirely distinct from a philosophy of the self. Because psychology and philosophy are distinct disciplines, Calkins stressed, self‐psychology is compatible with any philosophical understanding of the self, no matter how different or contrasting those understandings might be. Self‐psychology left the philosopher free to consider the self,

from the dualistic point of view as a form of reality coordinate with matter; or, from the standpoint of pluralistic idealism, the inter‐related system of selves may be treated as the final reality; or, finally, in terms of absolute idealism, reality may be conceived of as an absolute self, and the individual selves may be thought as related, by virtue of their manifestation of this Absolute.

(Calkins 1900, 492)

As we will see in the sections to come, Calkins herself considered the philosophical self in the final sense, and defended a metaphysical system that described how “the universe literally is one all‐including (and accordingly complete) self of which all the lesser selves are genuine and identical parts, or members” (Calkins 1931, 209). Her absolute personalism was strongly inspired by German idealism and the interpretation of this tradition by her other mentor from Harvard, Royce (Zedler 1995; McDaniel 2017). Calkins empathically maintained throughout her life that her endorsement of personalism did not affect her self‐psychology, as the two involved different disciplines of study.

## 3 The Pragmatisms: Psychological, Logical, and Metaphysical

In the first section, we examined Calkins's distinction between philosophy and psychology, as it provides the basis for addressing the first way in which she challenged the traditional narrative on pragmatism: interpreting pragmatism not solely as a logico‐philosophical movement, but as a tradition that includes psychological, logical, and metaphysical doctrines.

Before I start my analysis of Calkins's writings on pragmatism, it must be mentioned that Calkins's pragmatist writings occupy a relatively small part in her overall oeuvre. She provided her taxonomy of pragmatisms in a two‐page note that is included in the first four editions of *The Persistent Problems of Philosophy* (Calkins 1907, 1908, 1912a, 1917). This note, titled “note: pragmatism”, remained unchanged across all four editions and represents Calkins's first significant engagement with pragmatism. The note also represents her only printed engagement with pragmatism throughout most of her life, even though she taught several courses on pragmatism in her classroom at Wellesley (Calkins, “Philosophy Notes”, n.d.). Apart from this note, I have found just two other published writings that focus on pragmatism: a book review from 1912, and a new chapter added to the posthumously published fifth edition of *Persistent Problems*, which expands on the earlier “note”.

The relatively minor place that Calkins's pragmatist writings occupy in her oeuvre is an important historico‐philosophical fact, as it might lead one to question the significance of the movement—and of my analysis of it—for understanding her work. This doubt is mitigated, however, by the specific approach Calkins employs in these writings, which is to understand pragmatism within the framework of her distinction between science, specifically psychology, and philosophy. Calkins approaches many other subjects in a similar fashion, such as we might notice in her writings on ethics, and religion, which has led me to the conviction that this style of argument is central to our understanding of her thought (Kampen Forthcoming). I will thus proceed with my analysis of Calkins's discussion of pragmatism in the following two sections by focusing on the early “note: pragmatism”, while her later writings will be addressed in Section 5.

### 3.1 Psychological Pragmatism

Calkins's “note: pragmatism” begins with a definition of psychological pragmatism. She borrowed this definition from F. C. S. Schiller's *Humanism*, in which Schiller stated that pragmatism may be formulated as “the thorough recognition that the purposive character of mental life generally must influence and pervade also our most remotely cognitive activities” (Schiller 1903, as cited in Calkins 1907, 559). The influence of James may once again be noted in Calkins's decision to borrow from Schiller, as James wrote in his *Pragmatism* that “the best statements to begin with […] are F. C. S. Schiller's in his ‘Studies in Humanism’” (James 1907, viii). Endorsing Schiller's psychological definition of pragmatism, Calkins stressed that:

In this sense we all are, or ought to be, pragmatists; and we unquestionably owe a debt to contemporary pragmatists for laying stress on the noncognitive aspects of experience.

(Calkins 1907, 559)

Note, however, the conspicuous discrepancy between her endorsement and Schiller's original definition. While according to the latter, pragmatism describes how “the purposive character of mental life” influences “our most remotely cognitive activities,” Calkins redefines these activities as “the noncognitive aspects of experience” (Schiller 1903, 8; Calkins 1907, 559).

In *Humanism*, Schiller made it clear that with his definition, he was describing the “essential priority of action to thought” (Schiller 1903, 8). For Schiller and other pragmatists, the priority of action to thought included the belief that, at least partially, the traditional dualism between thought as cognitive and action as noncognitive needed to be reconsidered (Schiller 1903). Accordingly, Schiller argued that it was a mistake to understand the “cognitive,” that is, thought, as driving the “noncognitive,” that is, emotions and volitions (Schiller 1903, 82–83). For him, the new scientific findings of psychology and Darwinian theories of evolution had shown that individual behavior was related to “purposes”, which should be understood in the evolutionary sense of actions contributing to survival (Schiller 1903, 10). He argued that once we understand these actions, or “purposes”, as driving intelligent behavior, the traditional distinction between thought and action was no longer meaningful.

Given the importance of the priority of action to thought for Schiller, Calkins's reformulation of his remotely cognitive *activities* into noncognitive *aspects of experience* is striking. It raises the question of whether Calkins's understanding of psychological pragmatism also included Schiller's claim of the “essential priority of action to thought” (Schiller 1903, 8). I will return to this question in Section 5, the section in which I reflect on the development of Calkins's thoughts on psychological pragmatism. This initial description of psychological pragmatism suffices for the analysis of Calkins's taxonomy of pragmatisms vis‐à‐vis the traditional understanding of pragmatism as a logico‐philosophical movement, which I will now continue.

### 3.2 Logical and Metaphysical Pragmatism

Calkins stressed that logical pragmatism originated with Peirce's maxim that “our conception of the effects is the whole of our conception of the object” (Peirce 1878, as cited in Calkins 1907, 559). She argued that pragmatists attach two meanings to Peirce's maxim, namely:

(1) that the conception of the use, value, or consequences, of a reality form part of the conception of it [the object]; or (2) that the conception of a reality consists solely in the conception of its use or value.

(Calkins 1907, 559)

Calkins believed that in adherence to (1), “we are again practically unanimous”, that is, “to the adequate conception of object, situation, or truth there certainly belongs, as inherent part of it, a conception of its consequences” (Calkins 1907, 559). She argued that most objections to pragmatism are therefore directed to (2) and can be summarized as stating that the conception of an object “*is more than* a conception of its future, its results, its use, however truly the conception includes this awareness of practical consequences” (Calkins 1907, 560). To Calkins's discontent, the pragmatists had met this justified criticism by a defense of (1), and wasted “their time by reiterating the accepted statements that truth ‘makes a difference’ or that ‘personal attitudes or responses are real’” (Calkins 1907, 560).

Calkins went on to define metaphysical pragmatism as the logical consequence of (2) “that the conception of a reality consists solely in the conception of its use or value” (Calkins 1907, 559). From this strong form of logical pragmatism followed, according to her, “the doctrine that reality is to be defined only in terms of progressively unfolding experience and that there is, therefore, no ‘absolute’ or ‘complete’ reality” (Calkins 1907, 560). She added that “it is pragmatism of this sort only which necessarily involves pluralism” (Calkins 1907, 560).

With the remark that metaphysical pragmatism may justifiably be criticized, so that the conception of an object “*is more than* a conception of its future, its results, its use,” Calkins reveals that she rejected metaphysical pragmatism (Calkins 1907, 560). To review the scoreboard, we can conclude that she fully endorsed psychological pragmatism, partly endorsed logical pragmatism, but rejected metaphysical pragmatism.

This partial endorsement and her distinction between the different pragmatisms is consistent with her distinction between psychology and philosophy. For Calkins, metaphysical pragmatism included a claim about the nature of reality, namely “the doctrine that reality is to be defined only in terms of progressively unfolding experience and that there is, therefore, no ‘absolute’ or ‘complete’ reality” (Calkins 1907, 560). In Section 2, we have seen that Calkins stated “unambiguously that by ‘philosophy’ I mean ‘metaphysics’” (Calkins 1930b, 199). Her metaphysical pragmatism may accordingly be understood as “philosophical pragmatism”.

Calkins argued that philosophy is concerned with claims regarding the nature of experience, whereas psychology uses introspection to study experiences without inquiring into their philosophical nature. She legitimated the existence of both disciplines by arguing that their domains of study are and ought to be kept distinct. Thus, we might claim that Calkins does not endorse “philosophical pragmatism”, but, given the fact that philosophy must be distinguished from psychology, this still leaves her free to endorse psychological pragmatism and the weak form of logical pragmatism, considering that they do not necessitate any philosophical or metaphysical claims. In other words, Calkins's distinction between philosophy and psychology allowed her to reject one type of pragmatism while endorsing the others.

We will see in the following sections that Calkins's rejection of philosophical or metaphysical pragmatism followed from her disagreement with the pluralism that was involved in pragmatism. An understanding of this pluralism brings us to the second manner in which Calkins challenges the traditional narrative about pragmatism, by characterizing pragmatism as idealism. For Calkins did not simply argue that metaphysical pragmatism “necessarily involves pluralism”, but also that this pluralism must be understood as an idealistic pluralism called “pluralistic personalism” (Calkins 1907, 560).

## 4 Metaphysical Pragmatism as Idealism

Calkins's statement that metaphysical pragmatism necessitates a plural conception of reality is in itself not surprising, given that many pragmatists had defended pluralism at the time Calkins published *Persistent Problems*. In the 1898 lecture in which James introduced the term pragmatism, he had defended pluralism against Royce's monistic, or specifically, absolute conception of reality. In Section 2, I briefly discussed how Royce inspired Calkins's own absolute personalism. James, however, argued that Peirce's maxim revealed how “the régime of Royce's monistic Absolute Thought” would prove to be unnecessarily abstract (James 1898/2011, 59).

Seigfried has explained James's position by stressing that for him, reality is “always reality for someone” (Seigfried 2001, 19). Abstract concepts such as “the Absolute” could not be retraced to the lived experience of any individual, so that their meaning could not be explained by the pragmatic approach of describing their use or value. The inability to meaningfully speak of an “‘absolute’ or ‘complete’ reality” supported a plural conception of reality, in which, to use Seigfried's terms, “the plurality of interpretations cannot, in principle, be eradicated, although they may be mediated” (Seigfried 2001, 19; Calkins 1907, 560).

In the Introduction, we have seen Seigfried stressing the contrast between pragmatism and idealism. For her, it is a “longstanding misconception” to understand American pragmatism as the continuation of a metaphysical tradition, as “pragmatist philosophical analysis does not start with determining the nature of reality as such, the meaning of words, or with developing a logic of meaning, but with lived experience” (Seigfried 2001, 19). In this section, I will emphasize that Calkins saw the pragmatic focus on “lived experience” instead as a direct affirmation of an idealist understanding of reality, namely the understanding that “all reality is of the nature of consciousness” (Calkins 1907, 406). For her, James's position that reality is “always reality for someone,” meant that undetermined and therefore unmaterial selves were basic facts of reality, only importantly, as parts of the Absolute Self or Person (Calkins 1907, 1930b; Seigfried 2001, 19).

### 4.1 Pragmatism and Pluralistic Personalism

Calkins made no direct statements regarding idealism in the “note: pragmatism”. The interpretation of pragmatism as idealism follows from Calkins's categorization of her note on pragmatism under “Idealists” and “Pluralistic Personalists” (Calkins 1907, 557–560). Thus if we want to understand why Calkins chose to categorize her note in this way, thereby characterizing pragmatism as idealism, we must look at her description of the specific type of idealism that she ascribed to the pragmatists: pluralistic personalism.

Calkins described pluralistic personalism as the doctrine that “ultimate reality consists in the community, or society, of all related selves or spirits” (Calkins 1907, 413, 558). According to her, the pluralistic personalist acknowledged that “all reality is the nature of consciousness”, but also stated that:

consciousness […] is a conscious self or person, that is, a unique ‘real’ which is conscious and which may be regarded as including ideas, but which is more permanent than ideas are, and independent of them in a sense in which—on the contrary—they depend on it.

(Calkins 1907, 406–407)

The pluralistic personalist thus acknowledges a reality in which conscious states depend on a conscious self. These selves are independent from other selves, but are also of the same kind, for which the system can be described as *numerically pluralistic*, but *qualitatively monistic* (Calkins 1907, 413, 558).

To see why Calkins believed that pragmatism entailed this specific type of idealism, we must first remember her statement that it is metaphysical pragmatism “only which necessarily involves pluralism” (Calkins 1907, 560). Hence, only the metaphysical pragmatist must be considered a pluralistic personalist. This also follows from my interpretation that Calkins's taxonomy of pragmatisms is based on her thoughts on the distinctive subject matters of psychology and of philosophy. As idealism concerns a claim regarding the nature of reality, it belongs, according to Calkins, only to the domain of philosophy and not to psychology or any other science. As philosophy is synonymous with metaphysics, pluralistic personalism pertains only to metaphysical pragmatism.

So, why did Calkins consider the metaphysical pragmatist a pluralistic personalist? I have already stated that she provides no explanation of this in her note on pragmatism. However, she does consistently list two authors as both “pragmatists” and “pluralistic personalists” throughout the first four editions of *Persistent Problems*: James and Schiller. From the third edition onwards, Henri Bergson (1859–1941) is added to this list.

In the same year that Calkins published the third edition of *Persistent Problems*, she published an article in which she asserted that Bergson was viewed as a “temporalist” because “‘the fundamental principle’ of his whole philosophy is duration” (Calkins 1912a, 666). In opposing this interpretation, Calkins argued that Bergson was primarily a pluralistic personalist, because his philosophy had to be understood in the sense “that the reality, with which we are in immediate contact is—not duration—but the self which endures (*le moi qui dure*)” (Calkins 1912a, 666). From this article, then, we learn that Calkins believed Bergson to be a pluralistic personalist, because according to her, he considered “enduring selves” to be the most basic entities of reality.

To my knowledge, Calkins does not defend James's or Schiller's qualification as pluralistic personalists anywhere. This may be due to the fact that both philosophers had openly stated that consciousness is personal (James 1890/1950; Schiller 1903). As we have seen earlier, James's argument that “every thought tends to be part of a personal consciousness” had directly influenced Calkins's own psychological conception of self (James 1890/1950, Ch. 9, 225). Schiller, too, had acknowledged James's argument for the personal character of consciousness in his *Humanism* (1903, xvii–xviii). However, I believe there is one other important aspect that James's, Schiller's, and Bergson's philosophies share and which explains why Calkins considered them pluralistic personalists: they all defended free will.

Calkins articulates in her book on ethics, *The Good Man and the Good* (1918), that freedom must be contrasted with determinism:

freedom is often contrasted with mechanism or determination from without. Freedom, in this sense, characterizes the self—in particular, the active willing self—as opposed to the mere nature phenomenon…. Freedom in this sense is admitted not only by every ethical system but by every personalistic philosophy (183–184).

In this passage, Calkins argues that the free self is an undetermined self. To her, what is determined is ruled by the laws of nature. If selves have free will, this means that they are undetermined and are therefore “opposed to the mere nature phenomenon,” that is, the self considered as determined (Calkins 1907, 183–184). From Calkins's description that “freedom in this sense is admitted not only by every ethical system but by every personalistic philosophy”, we learn that the undetermined selves are, as she had stated in her description of pluralistic personalism, of “the nature of consciousness” (Calkins 1907, 406).

Although Calkins published this specific passage from her ethical book many years after her “note” on pragmatism, there are good reasons for believing that Calkins listed Bergson, James, and Schiller as personalistic pluralists precisely because their conception of reality included free, active, willing selves. In fact, she had already voiced the contrast between freedom and determinism in *Persistent Problems*, and in her article on Bergson's pluralistic personalism, she admitted that “the freely developing nature of the self as immediately realized by intuition, or instinct, is sharply contrasted … with the mechanical nature of the physical world as known to the intellect” (Calkins 1912a, 668).

The analysis of James, Schiller, and Bergson as pluralistic personalists in turn sheds light on Calkins's understanding of metaphysical pragmatism, or “the doctrine that reality is to be defined in terms of progressively unfolding experience” (Calkins 1907, 560). According to her interpretation, her mentor James as well as her contemporaries Schiller and Bergson all three understood “the progressively unfolding experience” as the experience of a self (Calkins 1907, 560). Because these three pragmatists also understood their selves to be free, Calkins viewed them as pluralistic personalists, that is, as adherents to a specific type of idealism in which selves are the most basic realities.

### 4.2 Pragmatism and the Personalistic Project

Now that we understand why Calkins characterized metaphysical pragmatism as a type of idealism, it is important to point out that her characterization hinges on a very specific interpretation of the philosophers cited above. James, Schiller and Bergson had voiced sufficiently clear objections to the idealistic tradition by the time Calkins published *Persistent Problems* (see e.g., Schiller 1891, 264−266; 1903, 197−198; Bergson 1896, 253−254; 1889, VII–VIII; James 1898/2011, 64–66; 1904, 533). I therefore feel forced to conclude that Calkins's specific interpretation of their thoughts testifies to what Orlo Strunk has called her “paradigmatic project” of a personalistic philosophy (Heidbreder 1972; Strunk 1972). To give this argument some additional evidence, let us close this section by taking a glance at Calkins's response to James's radical empiricism, many years after the first edition of *Persistent Problems*.

James had argued that radical empiricism included the claim that the most basic existent in the world is “pure experience”, which was “made of *that*, of just what appears, of space, of intensity, of flatness, of brownness, heaviness, or what not” (James 1904, 487). In two lectures presented in February 1923 and December 1923 and titled, respectively, “An Essential Factor in Every Truly Radical Empiricism” and “The Truly Radical Empiricism”, Calkins responded to his argument. What remains of Calkins's lectures are her handwritten notes, which require a significant degree of interpretation. One central objection to James's doctrine is clear, however, which is that his radical empiricism “falls short of being empiricism” (Calkins 1923a, 1923b). According to Calkins, a truly radical empiricism could make no claim regarding the existence of facts in experience, as the radical empiricist needs to hold the position “inextricable facts and no further” (ibid.). In other words, Calkins believed that when James claimed that experience is *all* that exists, he made a claim that he was not allowed to make based on experience alone (ibid.).

On the one hand, we may understand Calkins as effectively echoing a philosopher she had read very carefully, David Hume (1711–1776), and referring to his problem of induction: if James wanted to defend a true empiricism, he may list only instances and make no general claims. On the other, her criticism might be biased by her own understanding of “all that exists”, an understanding along the lines of “the principle of totality”, which James had formulated and rejected as early as the first version of “On Some Hegelisms” (James 1892a). What is important to our argument now, however, is that Calkins not only criticized James's argument that experience is all that exists, but also argued that what *is* gathered from experience is the conscious self, as she believed James had told her many years earlier. She stated that a “truly radical empiricism” should therefore acknowledge selves as the basic existent of reality (Calkins 1923b).

What Calkins's response to James's radical empiricism reveals is how she believed personalistic philosophy should be understood. For Calkins, personalism involved the argument that if consciousness is experienced as personal, this means that persons constitute the most basic existence. In other words, Calkins argued that the experience of the self in consciousness meant that there was a self that was fundamental to consciousness, and this conscious self was, in turn, fundamental to reality. She had already formulated this conclusion in her description of pluralistic personalism, stating that it included the conception that reality “is of the nature of consciousness”, and consciousness should be understood as “a conscious self…a unique ‘real’ which is conscious and which may be regarded as including ideas, but which is more permanent than ideas are, and independent of them in a sense in which—on the contrary—they depend on it” (Calkins 1907, 406–407).

Although Calkins made an effort to underscore personalistic philosophy by emphasizing the pragmatists as pluralistic personalists, she did not consider herself a pluralistic personalist, and thus, also not a metaphysical pragmatist. Throughout this paper, I have stated that Calkins endorsed an absolute and monistic personalism, the conception that “the universe literally is one all‐including (and accordingly complete) self” (Calkins 1930b, 209). In the last and final section of this paper, I look at Calkins's two late writings on pragmatism to relate Calkins's rejection of pluralistic personalism and metaphysical pragmatism to her endorsement of both absolute personalism, and, as I discussed in Section 3, psychological pragmatism.

## 5 Calkins's Endorsement of Absolute Personalism and Psychological Pragmatism

After publishing her “note: pragmatism” in the first four editions of *Persistent Problems*, Calkins only directly discussed pragmatism in a book review of Addison Webster Moore's *Pragmatism and Its Critics* (1912b) and in a new chapter added to the posthumously published fifth edition of *Persistent Problems* (1936). In this last section, I will discuss these two writings to examine Calkins's endorsement of both absolute personalism and psychological pragmatism. My argument is that both endorsements can be explained by psychological and philosophical commitments of Calkins that, in turn, might raise problems for her distinction between the two disciplines, as well as raise questions for the broader relation between pragmatism and metaphysics.

### 5.1 Absolute Personalism vis‐à‐vis Metaphysical Pragmatism

Calkins rejected metaphysical pragmatism and pluralistic personalism because she defended a *numerical and qualitative monism* called “absolute personalism”. She explained absolute personalism by stressing that it shared with pluralistic personalism, “the doctrine that the universe is ultimately consciousness, and that consciousness means selves or Self” (Calkins 1907, 418). However, it differed from pluralistic personalism in the sense that “ultimate reality is no system, community, or kingdom of selves, but *a Self* (Calkins 1907, 418; emphasis added).

Kris McDaniel has demonstrated that there are many interesting things to say about Calkins's absolute personalism (McDaniel 2017, 2019). He contends that a central argument in her defense of absolute personalism is her claim that “the Absolute serves as the ground of truth” (McDaniel 2019, 580). Calkins makes this claim in her book review of Moore's *Pragmatism and Its Critics* (1912c).

In his book, Moore had stated that Peirce's maxim, and James's interpretation of it, were preferable to other truth‐theories as they avoided any metaphysical parlance regarding universal truths, or the Absolute. Reiterating statements from James's lecture on pragmatism, Moore stressed that the pragmatist saw no connection between truth theories that explained truth as a correspondence relation between an individual and the Absolute and the actual practice in which we usually determine truth (Moore 1910, 192–194). Moore noted that if the experienced results of a belief could explain “just by what character judgements are true”, it was “in the thick of logic”, so that “it is difficult to see just what there is left to be explained or interpreted by some other *kind* of logic” (Moore 1910, 194).

Calkins responded to these statements in her review by admitting that the pragmatist theory of truth is indeed “the most convenient ‘test’ of truth” (Calkins 1912b, 225). However, she argued that it could only strictly be called “an indication of the truth” (Calkins 1912b, 225). The only way in which we could actually account for truth was by means of the existence of an “Absolute Person” (Calkins 1912b; Royce 1895/1969a; McDaniel 2017, 2019). While up to this point, we have mainly noticed James's influence on Calkins thought, we must here acknowledge the considerable influence of Royce, her other mentor. Indeed, in her book review, Calkins explained that her argument for the existence of an Absolute Person was based on Royce's argument concerning the Absolute Self and truth (Calkins 1912b, 225).

If we look at the basis for Royce's well‐known argument on truth—and error—we find that all truth is “a truth somewhere experienced” (Royce 1885, 1895/1969, 379; Dunham 2020). Royce argued that if there existed no absolute or complete reality, but only a plurality of truths, then this would itself have to be an absolute truth. But if the denial of a complete reality was a truth, this raises the rhetorical question: “for whose experience is it to be a truth?” (Royce 1895/1969, 378−379). For Royce, as for Calkins, such an experience could only be of a person capable of comprehending the complete reality— a reality that was hypothetically denied—so that the denial of a complete reality required an Absolute Person (Royce 1895/1969; Dunham 2020).

Calkins's acceptance of this argument meant that she believed that the existence of an Absolute Self was required to secure the absolute truth of any statement, even if the statement was the pragmatist's claim “that there is “no ‘absolute’ or ‘complete’ reality” (Calkins 1907, 560). Considering that the denial of an absolute reality belonged to the description of metaphysical pragmatism, that is, “the doctrine that reality is to be defined only in terms of progressively unfolding experience and that there is, therefore, no ‘absolute’ or ‘complete’ reality,” this in turn explains why Calkins considered herself an absolute personalist, rather than a pluralistic personalist (Calkins 1907, 560).

### 5.2 The Reformulation of Metaphysical Pragmatism to Epistemological Pragmatism

Calkins developed and revised her interpretation of Royce's argument on truth for her fifth edition of *Persistent Problems*, even though she died before she could publish it. In this posthumously published fifth edition of her philosophical treatise, the distinction between the different pragmatisms remains in place, but it is now simplified to a distinction between psychological pragmatism and “epistemological pragmatism”, which Calkins both considered to have historically followed from the logical pragmatism described by Peirce's maxim (Calkins 1936, 399).

In her reformulation of metaphysical pragmatism as epistemological pragmatism, Calkins shifted the emphasis from pragmatism as based on pluralism to pragmatism based on claims regarding the existence of truth. She consequently described epistemological pragmatism as “the theory that there is no ‘fixed Truth already in existence’,” referring to Dewey's *Reconstruction in Philosophy* (1920) as well as to James's *Pragmatism* (1907). Consequently, her rejection of epistemological pragmatism has the same form as her rejection of metaphysical pragmatism in the book review, but the way the criticism is formulated focusses on the presupposition of truth, rather than the presupposition that there is “no ‘absolute’ or ‘complete’ reality” (Calkins 1907, 560). In the fifth edition of *Persistent Problems*, Calkins invoked the words of the philosopher Edward Gleason Spaulding (1873–1940), and stressed that “‘the pragmatic theory of truth…is advanced as one that is true absolutely and therefore… absolute truth is presupposed in pragmatism’” (Calkins 1936, 405).

By formulating the criticism in an epistemological sense rather than a metaphysical sense, Calkins focused on how pragmatism presupposed absolute truth rather than an Absolute Self. However, this shift in focus was not inspired by doubts regarding the existence of the Absolute Self. In this same fifth edition of *Persistent Problems*, Calkins still fiercely defended her absolute personalism. The reason that Calkins wanted to sever the interpretive ties between pluralism and pragmatism was because she believed by the last edition of *Persistent Problems,* that pragmatists had mainly focused on criticizing the absolute conception of reality, while they had failed to defend the epistemological claim that “absolute truth is presupposed in pragmatism” (Calkins 1936, 405).

### 5.3 Psychological Pragmatism and the Distinction Between Philosophy and Psychology

Now that we have seen why Calkins favored absolute personalism over metaphysical pragmatism, or rather, by that time, epistemological pragmatism, we will close this paper with a final analysis of the type of pragmatism that she did endorse: psychological pragmatism. In the fifth edition of the *Persistent Problems*, Calkins holds on to the argument that her rejection of epistemological pragmatism did not involve a rejection of psychological pragmatism (Calkins 1936). As we have seen above, I explained that this partial endorsement of pragmatism resulted from her ideas about the distinction between philosophy and psychology. She reiterated this distinction in the fifth edition of *Persistent Problems* and argued that psychological pragmatism was compatible with different philosophical systems, because it was “metaphysically neutral” (Calkins 1936, 398–399).

I also raised an issue earlier regarding Calkins's support of psychological pragmatism which we are now able to resolve. The issue was whether Calkins fully endorsed Schiller's specific understanding of pragmatism. In the first four editions, Calkins borrowed Schiller's definition of pragmatism to describe psychological pragmatism. Yet she rephrased his definition in her subsequent sentence, reformulating his “remotely cognitive *activities*” into “noncognitive *aspects of experience*” (Schiller 1903, as cited in Calkins 1907, 559). I pointed out that for Schiller and the “functional pragmatists,” the understanding of activities as cognitive was important, because it stemmed from a reconsideration of the traditional dualism between thought and action. They understood pragmatism as criticizing the established idea that it was the “cognitive,” that is, thought, that was driving the “noncognitive,” that is, emotions and volitions (Schiller 1903, 82–83).

However, it seems that what was basic for Calkins was the *introspected experience* of the self, not the *actions* or “purposes” that Schiller and the other pragmatists had mentioned as being prior. I am convinced that this aspect of the movement was not included in either Calkins's early or late support of psychological pragmatism. In the fifth edition of *Persistent Problems*, Calkins changed her definition of psychological pragmatism by citing Dewey instead of Schiller. Yet, here too, she rephrases Dewey's statement in a way that upholds the traditional distinction between thought and action. She states, after Dewey, that in psychological pragmatism “the senses lose their place as gateways of knowing to take their rightful place as stimuli to action” (Dewey 1920, in Calkins 1936, 398–399). Then she writes:

… humanists and [psychological] pragmatists make common cause against those psychologists, economists, and moralists, and against those philosophers as well, who have seemed to conceive human beings as purely perceiving and “rational animals” rather than as the impulsive, feeling, and active beings which they are.

(Calkins 1936, 399)

Dewey's statement that the senses should be understood as stimuli to action is here rephrased so as to create a distinction between human beings as “rational animals,” and “impulsive, feeling, and active beings” (Calkins 1936, 399).

So why did Calkins seem to hold on to the traditional distinction between thought and action, while the contemporaries she quoted held a different view? One might conjecture that she believed that the criticism of the distinction between thought and action was an unwarranted intrusion of metaphysical thinking into the realm of psychology. But I believe that there is another explanation for Calkins's rephrasing, one that in fact raises the question of whether she herself did not cross the bridge between metaphysics and psychology. The reason that Calkins held on to the traditional distinction between thought and action had to do with the fact that she believed experience to reveal a solely mental reality. She could not acknowledge action as essentially prior, because for her, all was thought.

In *A First Book in Psychology* (1910), Calkins stated that it was important to distinguish between philosophy and psychology, for “the psychologist, it must be confessed, is sometimes tempted to overstep the border” (2). Yet it seems that she crossed this border herself in her conviction that experience is essentially mental, so that psychological facts are, strictly, psychic facts. At the end of her life, Calkins formulated the relation between experience and reality as follows:

… what any man unchallengeably knows—what he knows, in other words, without any element of hypothesis—is himself and his experiencing, clearly a mental reality.

(Calkins 1931, 205)

Admittedly, this citation comes from Calkins's philosophical writing, so that we might still consider this a philosophical statement rather than a crossing of domains. However, it seems that this argument on the relation between experience and reality also influenced Calkins's psychological thought. For instance, from notes for a class on self‐psychology in 1926, it emerges that, around that time, Calkins indeed taught the *psychological* self to be “nonmaterial” (Calkins 1926). Furthermore, a former student of Calkins, Josephine Nash Curtis, published a critical discussion of self‐psychology in 1915 in which she argued that by intentionally omitting the body and soul from the conception of self, Calkins could no longer claim that she understood the self in the sense of “what the plain man means by self” (Calkins 1906, 63; Kampen Forthcoming). According to Curtis, common people would include either the body or the soul in their conception of self, so that the intentional omission of these aspects revealed Calkins's metaphysical or philosophical commitments (Curtis 1915, 74).

It seems thus that Calkins supported a type of psychological pragmatism that emphasized selves as “impulsive, feeling, and active beings”, yet she also considered these beings as solely psychical, instead of, for example, psychophysical. Calkins's psychic selves fitted within her metaphysical framework of individual selves as parts of an Absolute Self, a framework that was perhaps less distinct from her psychology than she intended.

If we compare Calkins's specific understanding of psychological pragmatism to the traditional narrative on pragmatism with which we started this paper, we might conclude that she contributed to the “longstanding misconception” of understanding pragmatism as the continuation of the American idealist tradition (Seigfried 2001, 13). Yet to reduce Calkins's interpretation of pragmatism to a misconception is, to my mind, neither historically nor philosophically warranted. We might instead wish to draw two conclusions that offer productive directions for future investigation. First, Calkins's interpretation teaches us how, during the early days of pragmatism, its reception included a variety of interpretations from significant scholars that diverge from the general narrative that we embrace nowadays. Second, Calkins's attempt to distinguish between the different forms of pragmatism invites reflections on her other distinction, namely that between psychology and philosophy, or, in Calkins's terms, psychological and metaphysical subject matters. As we have seen, Calkins may have inadvertently overstepped her own border. However, her taxonomy of pragmatisms invites us to wonder whether, in their revolt against metaphysics, the pragmatists she discusses not also made metaphysical claims, for instance that reality is numerically plural, or consciousness psychophysical.

## 6 Conclusion

As we have seen in this article, Calkins made a sustained effort to separate the new science of psychology from philosophy. Her thoughts on the proper subject matters and methodologies of these disciplines resound in her writings on American pragmatism, which she believed included psychological, logical, and metaphysical doctrines. Calkins's conviction that pragmatism was a type of idealism was also reflected in her understanding of the metaphysical pragmatists as being pluralistic personalists.

With her characterization of metaphysical pragmatism as pluralistic personalism, Calkins took these philosophers not only to hold that “reality is the nature of consciousness”, but also that consciousness should be understood as a conscious self, a “unique ‘real’ which is conscious” (Calkins 1907, 406–407). This philosophical self was more permanent than ideas are, and in fact, all conscious ideas depended upon it.

Although Calkins was a personalist herself, she rejected the pluralism of the metaphysical pragmatists on the grounds that she remained unconvinced by the pragmatic theory of truth. Calkins followed her mentor Royce in stating that (1) truth is experienced by a self, and (2) even the denial of an Absolute must itself be taken as an absolute truth, so that for this truth to be experienced it required the existence of an Absolute Self. In her later writings, she would reformulate this argument to state simply: “absolute truth is presupposed in pragmatism” (Calkins 1936, 405).

Calkins emphasized in her early and late writings that her rejection of metaphysical pragmatism did not involve a rejection of psychological pragmatism. This argument was again based on her distinction between philosophy and psychology. Calkins believed that only philosophy made metaphysical claims, so that her rejection of the metaphysical claim of pragmatism did not involve a rejection of the psychological claim of pragmatism. However, Calkins's arguments regarding the relation between experience and reality question whether she, in fact, endorsed a psychological pragmatism that was metaphysically neutral.

Calkins's statements on experience and reality seem to prescribe a metaphysical conception of reality that was thoroughly mental in nature, which explains her struggle to commit to the psychophysicalism that accompanied some of the other pragmatists' theories. It also invites deeper reflection on whether pragmatism and metaphysics—or even psychology and philosophy—can ever be clearly separated. Put differently, the traditional narrative on pragmatism rightly stresses the pragmatists' criticism of the metaphysical tendencies of the early American idealists. However, in their revolt, the pragmatists might have implicitly made their own metaphysical claims, by stating that the universe consisted of a plurality of beings, or that these beings may best be understood as psychophysical.

## Conflicts of Interest

The author declares no conflicts of interest.

## Data Availability

Data sharing not applicable to this article as no data sets were generated or analyzed during the current study.
